# Crystallization of ZnO thin films without polymer substrate deformation *via* thermal dissipation annealing method for next generation wearable devices†

**DOI:** 10.1039/d0ra09869h

**Published:** 2021-01-04

**Authors:** Dongwan Kim, Jae-Young Leem

**Affiliations:** Department of Nanoscience & Engineering, Inje University 197, Inje-ro Gimhae-si Gyeongsangnam-do 621-749 Republic of Korea jyleem@inje.ac.kr

## Abstract

The synthesis method of transparent and flexible ZnO thin films is currently considered the most important factor for the fabrication of next generation wearable devices. To fabricate transparent and flexible devices by using sol–gel spin-coated ZnO thin films, an annealing step is necessary; however, annealing processes at high temperatures decompose polymer substrates due to their low melting temperature. It was found that sol–gel spin-coated ZnO thin films can be crystallized through the mobility difference of ZnO molecules placed at the surface of ZnO thin films. Especially, ZnO thin films can be annealed at high temperature (above 500 °C) by using a thermal dissipation annealing (TDA) method without the deformation of the polymer substrate. A transparent and flexible ultraviolet photodetector based on ZnO thin films annealed with the TDA method exhibited fast rise and decay time constants, a high on/off current ratio, and reproducible photocurrent characteristics. Thus, these results indicated that the TDA method is a feasible alternative route for the fabrication of next generation wearable devices.

## Abstract 2

Thermal dissipation annealing method is an effective way of fabricating transparent and flexible optoelectronics for next generation wearable devices.

## Graphical abstract

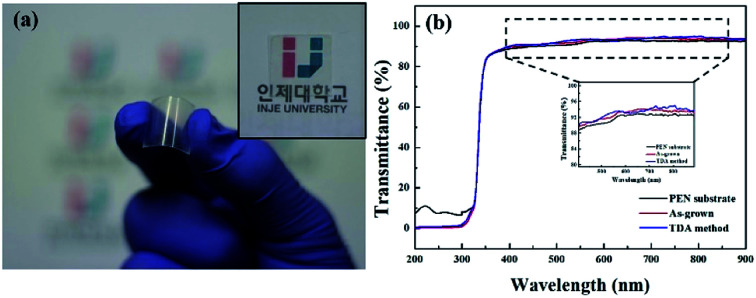

## 1. Introduction

Transparent and flexible electronics are one of the most essential technologies for the next generation of optoelectronic devices, such as transparent displays, ultraviolet (UV) photodetectors, gas sensors, solar cells, and wearable devices.^1–7^ In particular, UV photodetectors have attracted considerable research interest in the last few years because of their great potential and commercial impact in a wide variety of fields, such as secure space-to-space communications, pollution monitoring, water sterilization, flame sensors, and missile plume detection applications.^8,9^ Various semiconductors, such as Si, ZnS, and ZnO, have been used for UV photodetectors. Among them, Si-based UV photodetectors exhibit a fast response; however, the narrow band gap of Si deteriorates the sensitivity and selectivity of low energy photons (visible and infrared (IR)) light.^11^ Therefore, when Si-based UV photodetectors are used, a complex filter is required to avoid noise related to low electron energies. Additionally, for the accurate detection of Si-based UV photodetectors, an ultrahigh vacuum and high voltage are required.^10^ In the case of ZnS-based UV photodetectors, a complex filter is not required due to the wide band gap of ZnS (3.91 eV), but ZnS-based UV photodetectors show weak value and poor stability of photocurrent, and they only react to UV light with wavelengths shorter than 335 nm.^12^ In contrast, ZnO-based UV photodetectors are free from the requirement of complex filter due to their wide direct band gap of 3.37 eV and large exciton binding energy of 60 meV.^13–15^ Additionally, ZnO is a suitable material for transparent and flexible optoelectronics owing to its high electron mobility and high transparency in the visible region. To fabricate ZnO based UV photodetector, various deposition method, such as metal–organic chemical vapor deposition, molecular beam epitaxy (MBE), pulsed laser deposition, sol–gel spin-coating method, and hydrothermal method, have been used. Among them, the deposition of ZnO thin films by using the sol–gel spin-coating method has a variety of advantages, such as simplicity, easy control of the doping level, straightforward solution concentrations, the production of homogeneous films, and feasibility of large-area deposition without the use of expensive and complicated equipment that is required with other methods.^16–19^ However, despite of these advantages, the deposition of ZnO thin films by using sol–gel spin-coating method has a critical drawback: crystallization of amorphous state ZnO thin films. When ZnO thin films are deposited with the sol–gel spin-coating method, the ZnO thin films are amorphous.^20,21^ Amorphous ZnO thin films have a worse crystallinity than polycrystalline ZnO thin films due to the elevated grain boundary ratio and large number of point defects; these drawbacks deteriorate the performance of ZnO-based transparent and flexible optoelectronic devices. Therefore, sol–gel spin-coated ZnO thin films must be crystallized to improve the performance of ZnO-based transparent and flexible optoelectronic devices. For a long time, many researchers have attempted to effectively anneal sol–gel spin-coated ZnO thin films. Li and Gu *et al.* reported the effects of annealing temperature on structural, optical, and UV photoresponse properties of ZnO thin films derived by the sol–gel spin-coating method and demonstrated that the annealing process can effectively enhance the crystallinity and UV photoresponse properties of ZnO thin films by decreasing the number of defects in the ZnO lattice.^22,23^ Additionally, the excimer laser and electron beam annealing method can effectively anneal ZnO thin films at low temperatures, and our group has devised a novel annealing method named the counterpoise-assisted annealing (CAA) method, in which an external force is applied to the thin films by placing a counterpoise on the ZnO thin films, to anneal the sol–gel derived ZnO thin films without bending of polymer substrates.^24–26^ However, to fabricate ZnO-based transparent and flexible optoelectronic devices, ZnO thin films have to be deposited onto transparent and flexible polymer substrates that exhibit high transparency in the visible region and high flexibility at room temperature. For example, the use of polyethylene terephthalate (PET) and polyethylene naphthalate (PEN) and annealing processes at high temperatures are not possible due to low glass transition temperature (130 °C) and melting point (200 °C) of polymer substrates. Additionally, annealing with electron beam and excimer laser processes is unsuitable because the high energy density of the excimer laser and electron beam deforms the polymer substrates. In the case of the CAA method developed by our group, a transparent and flexible polymer substrate can be used, but substrate options are restricted to muscovite mica and polyimide, which exhibits a higher melting point (400 °C) than PEN and PET. Therefore, to fabricate transparent and flexible optoelectronic devices using polymer substrates, it is necessary to develop a new annealing method that can anneal sol–gel spin-coated ZnO thin films without melting or deforming the substrate.

In this study, we developed a novel annealing method, the thermal dissipation annealing (TDA) method, that eliminates the thermal energy from substrates through a thermal dissipation system combined with an IR lamp and cold plate to prevent melting of polymer substrates. Furthermore, to confirm the annealing efficiency and heat transfer rate of the TDA method, we deposited ZnO thin films onto the PEN substrates sol–gel spin-coating method and annealed the ZnO thin films at 500 °C with TDA methods. Additionally, we fabricated transparent and flexible ZnO-based UV photodetectors to suggest the feasibility of the TDA method in next-generation wearable optoelectronics devices.

## 2. Experimental

### 2.1 Preparation for ZnO thin films

ZnO thin films were deposited onto PEN substrates by using the sol–gel spin-coating method and annealed by using the TDA method. A ZnO sol–gel solution was prepared by dissolving zinc acetate dihydrate ([Zn(CH_3_COO)_2_·2H_2_O], guaranteed reagent, ≥99.0%, JUNSEI) and monoethanolamine (MEA)([NH_2_CH_2_CH_2_OH], ACS reagent, ≥99.0%, Sigma-Aldrich) in 2-methoxyethanol ([C_3_H_8_O_2_], guaranteed reagent, ≥99.0%, JUNSEI) to deposit ZnO thin films onto Si and PEN substrates. MEA was used to stabilize the solution and enhance the solubility of the precursor salt. The zinc acetate/MEA molar ratio equalled 1 : 1, and the concentration of the ZnO precursor solution was 0.5 M. The prepared mixture was stirred at 60 °C for 2 h to obtain a clear homogeneous solution that was used as a coating source after being cooled to room temperature. After preparing the sol–gel solution, the PEN substrates were cleaned by ultrasonication in isopropyl alcohol (C_3_H_8_O, guaranteed reagent, ≥99.7%, JUNSEI) for 20 min, rinsed with distilled water for 2 min and dried with nitrogen gas (99.9999%).

### 2.2 Deposition of ZnO thin films

The ZnO thin films were deposited by using sol–gel spin-coating method. The prepared sol–gel solution was deposited onto the cleaned PEN substrates and spin-coated at 2000 rpm for 20 s. The spin-coated ZnO thin films were preheated at 150 °C for 10 min in an oven. The spin-coating and preheating processes was repeated nine additional times. Subsequently, the obtained ZnO thin films were annealed at 500 °C for 1 h by using the TDA method.

### 2.3 Characterization

The morphology of the ZnO thin films was measured by FE-SEM (TESCAN NIRA3LM) on an instrument with an accelerating voltage of 30 kV. The crystal phases were analysed by XRD (PANalytical X'Pert Pro) using a Cu-Kα radiation source (*λ* = 0.15406 nm) at an accelerating voltage of 40 kV. The transparency of the ZnO thin films was measured by using UV-visible spectroscopy (Thermo Scientific Evolution 220). In addition, the photoresponse changes were measured at a bias voltage of 0.1 V using a UV light (*λ* = 365 nm) with a power density of 10 mW cm^−2^.

## 3. Results and discussion

**Fig. 1:**
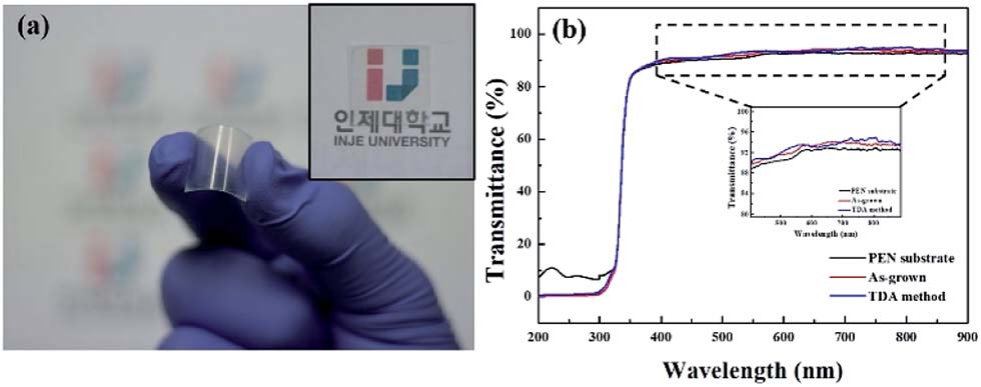
(a) Photographic image of the transparent and flexible UV photodetector fabricated by using TDA annealed ZnO thin films and (b) transmittance spectra in the wavelength range from 200 to 900 nm for ZnO thin films deposited onto PEN substrate.

Fig. 1(a) shows a photograph of the sol–gel spin-coated ZnO thin films on the PEN substrate, which confirmed that the ZnO thin films annealed with the TDA method had a high transparency. Despite the annealing process at a high temperature, the PEN substrate maintained its flexibility and transparency without melting or deforming. Fig. 1(b) shows the optical transmittance of the PEN substrate and ZnO thin films deposited onto the PEN substrate. The PEN substrate presented approximately 90% transmittance at the wavelengths ranging from 400 nm to 900 nm. After the deposition and annealing process, the ZnO thin films deposited onto the PEN substrate had a similar transmittance compared with that of the bare PEN substrate, indicating that the deposition and annealing process barely influenced the transparency of the ZnO thin films. However, when a sol–gel spin-coated ZnO thin film was annealed in a furnace, the PEN substrate was deformed and transformed to a powder, and when a sol–gel spin-coated ZnO thin film was annealed with an IR lamp, the PEN substrate melted due to the low melting temperature (185–200 °C) of the PEN substates, as shown in Fig. S1,† which shows photographs of the ZnO thin films after the annealing process in a furnace and with an IR lamp.

**Fig. 2:**
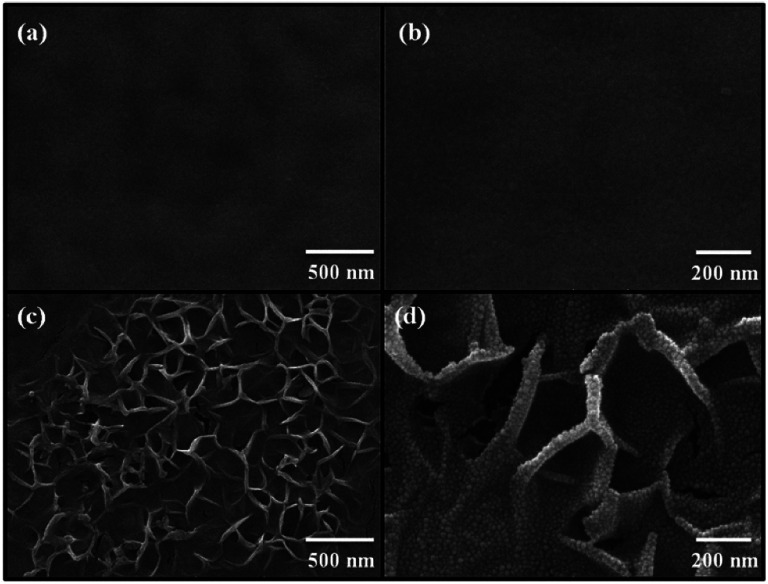
FE-SEM images of (a), (b) non-annealed ZnO thin films and (c) and (d) ZnO thin films annealed by using TDA method.

**Fig. 3:**
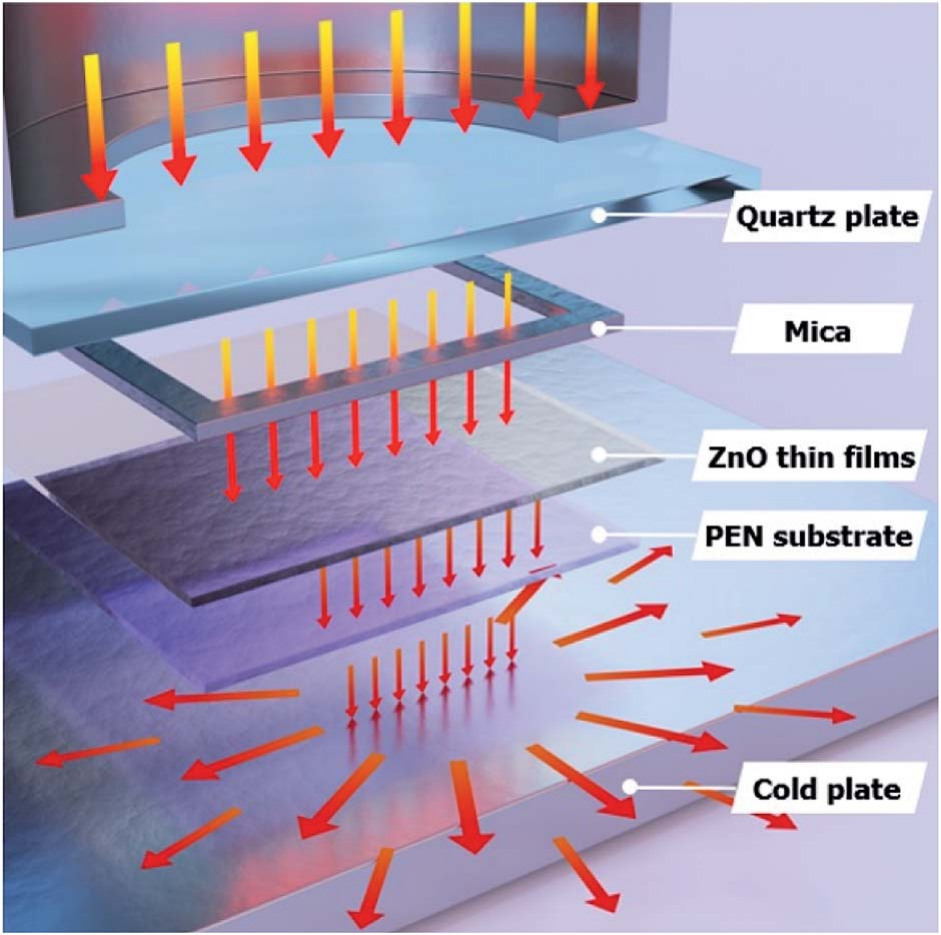
Schematics images for annealing process of TDA method.

Fig. 2 shows the FE-SEM images of non-annealed and annealed ZnO thin films by using the TDA method. The wrinkled network structures are observed in the non-annealed ZnO thin films and the graphene-like nanosheets were observed in the ZnO thin films annealed by using the TDA method. Herein, the main reason of the graphene-like nanosheet formation with the TDA method is due to the mobility difference of ZnO molecules consisted of ZnO thin films. In general, the surface molecules of ZnO thin films deposited onto a low-temperature substrate exhibit a short migration length due to the low temperature of the substrate, whereas when thin films are deposited on a high-temperature substrate, the surface molecules of the thin films exhibit enhanced diffusion.^27^ Chaâbane *et al.* reported that a low substrate temperature increases the density of thin films composed of nanocrystals due to an increase in the residence time.^28^ With respect to the TDA method, the ZnO thin films deposited onto a PEN substrate are placed on the cold plate for 30 s to decrease the temperature of the PEN substrate and a thin mica plate with a hollow square at its center is placed on the ZnO thin films, as shown in Fig. 3. As the annealing proceeded, some of the thermal energy is absorbed into the surface of ZnO thin films and used to crystallize the amorphous ZnO thin films, whereas the thermal energy reached at the PEN substrate is directly dissipated through the cold plate. At this point, there was a temperature difference between the surface and bottom of the ZnO thin films that changed the mobility of the ZnO molecules at the surface and bottom of the wrinkled network structures. In other words, the ZnO molecules at the surface of the wrinkled network structures had an elevated mobility owing to the large amount of thermal energy and high temperature and moved to the bottom of the wrinkled network structures to reach a thermal equilibrium state. On the other hand, the ZnO molecules at the bottom of the wrinkled network structures, which had a decreased mobility owing to a small amount of thermal energy and low temperature, did not move to the surface of the wrinkled network structures. As the annealing continued, additional ZnO molecules the surface moved to the bottom of the wrinkled network structures, and the thickness at the edge of the wrinkle network structures became thinner than that of the center of the wrinkled network structures due to the increased amount of ZnO molecule movement. At the end of the annealing process, the thermal energy required for crystallization was transferred to the ZnO molecules placed at the bottom of the wrinkled network structures through the relatively thin wrinkled network structure at the edge, which caused the crystallization of the ZnO thin films at the bottom of the wrinkled network structures. As a result, the thermal dissipation system of TDA method enables the crystallization of ZnO thin films without substrate deformation at the high temperature (Fig. 1).

**Fig. 4:**
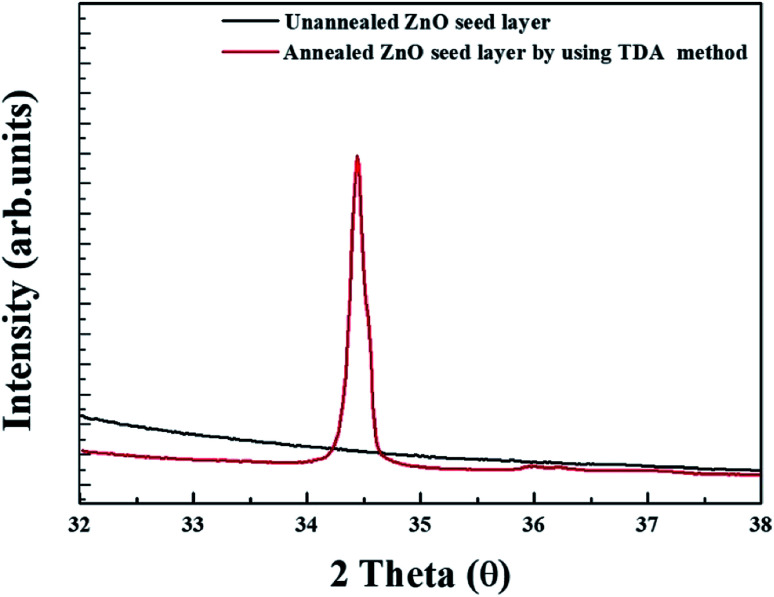
XRD patterns of unannealed and annealed ZnO thin films.

**Fig. 5:**
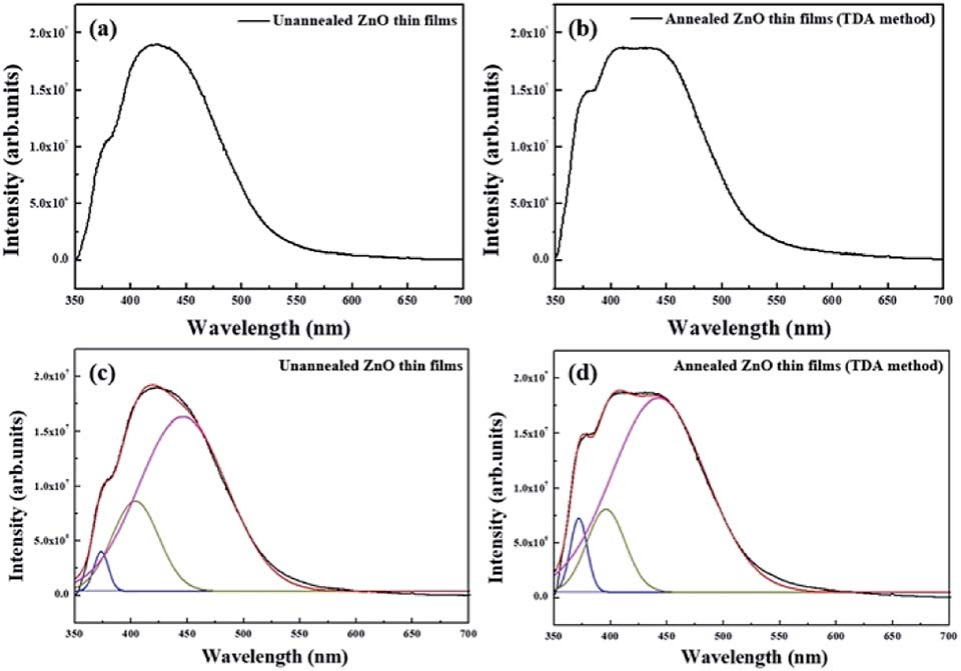
PL spectra of (a) non-annealed and (b) annealed ZnO thin films by using TDA method and Gaussian-fitting analysis of the PL spectra of (c) non-annealed and (d) annealed ZnO thin films by using TDA method.

Fig. 4 shows the XRD patterns of unannealed and annealed ZnO thin films. As shown in Fig. 5, no diffraction pattern was observed in the unannealed ZnO thin films and it means that the crystal state of the sol–gel spin-coated ZnO thin films is amorphous state. However, a strong diffraction peak, which is diffraction peak from ZnO (002) plane, and a weak diffraction peaks, which is attributed to the diffraction peak from ZnO (101) plane, are observed at 34.6° and 36.4° in the annealed ZnO thin films, indicating that the sol–gel spin-coated ZnO thin films can be crystallized by using the TDA method.^29^

**Fig. 6:**
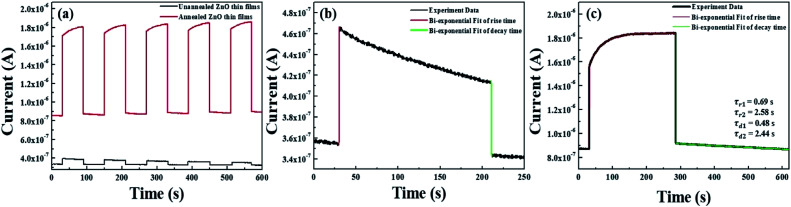
(a) Time-dependent UV photoresponse of non-annealed ZnO thin films and annealed ZnO thin films and rise and decay of photoresponse curves for (b) non-annealed ZnO thin films and (c) annealed ZnO thin films by using TDA method.

Fig. 5(a) and (b) show the PL spectrum of the non-annealed ZnO thin films and annealed ZnO thin films annealed with the TDA method, respectively. The PL spectrum of the non-annealed ZnO thin films was composed of two bands with peaks at approximately 380 nm and 425 nm, and the PL spectrum of ZnO thin films annealed by using the TDA method was composed of two bands with peaks at approximately 380 and 420 nm. The peak observed at approximately 380 nm was due to the near-band-edge (NBE) emission of the ZnO thin films, which was attributed to the recombination of the free excitons.^30^ However, the origin of the peaks observed at approximately 420 nm and 425 nm was unclear. To determine the origin of these peaks, we fitted the PL spectra of the ZnO thin films by using Gaussian functions, as shown in Fig. 6(c) and (d). Due to pitting, the best fit of the PL spectra was obtained with three Gaussian functions that had peaks centered at 380 nm, 400 nm, and 440 nm. The First was a NBE emission centered at 380 nm, which was attributed to the recombination of free excitons in the ZnO, and the intensity of the NBE emission increased by annealing the ZnO thin films with the TDA method. The second was a broad emission centered at approximately 400 and 440 nm, which did not undergo a change in emission intensity and likely originated from the PEN substrate. This increase in the NBE emission intensity and lack of a DL emission corresponded to the PL spectrum change of the ZnO thin films deposited onto the Si substrate and the clearly enhanced intensity of NBE emission of the ZnO thin films indicated improvement of crystallinity of the ZnO thin films.^31^

To confirm the fabrication possibility of transparent and flexible optoelectronics through TDA method, we fabricated metal–semiconductor-metal (MSM) UV photodetectors through the deposition of an indium electrode onto the ZnO thin films and measured the photoresponse of the ZnO thin films by periodically turning a UV light with a power density of 10 mW cm^−2^ on and off (*λ* = 365 nm) in air. As shown in Fig. 6(a), it can be seen that the photocurrent increased sharply as soon as the ZnO thin films were exposed to UV light and dramatically decreased after the light was turned off; the ZnO thin films also demonstrated excellent stability and reproducibility. However, with respect to the unannealed ZnO thin films, despite of the highly fast photoresponse speed, the current increment was very slight and it is due to the poor crystallinity and defect sites of amorphous ZnO thin films which bound the free electrons. In addition, the photocurrent value of the ZnO thin films annealed by the TDA method clearly increased by a factor of 5.29 compared to that of the unannealed ZnO thin films under the same measurement conditions (power density of UV light, temperature, and humidity). This increment difference of photocurrent can be demonstrated by following mechanism. When the ZnO thin film-based UV photodetector is exposed to air in the dark, the oxygen molecules are adsorbed on the ZnO thin films surface and capture free electrons from the ZnO thin films, forming a depletion region near the surface and causing a decline in conductivity of the ZnO thin films (O_2_ + e^−^ → O_2_^−^).^32–34^ When the ZnO thin films based UV photodetector are exposed to UV light, electron–hole pairs generated, and some photogenerated holes migrate to the surface of the ZnO thin films to combine with electrons that capture the oxygen molecules, resulting in a reduction in the depletion barrier thickness (h^+^ + O_2_ → O_2_).^35,36^ At the same time, the photogenerated electrons move to the electrode based on the bias voltage, resulting in a photocurrent increase. When the UV light is turned off, the photogenerated electron–hole pairs instantly recombine with each other and the readsorption of the oxygen molecules occurs on the surface of the ZnO thin films, resulting in a decrease in the photocurrent.^34^ Therefore, in the case of the non-annealed ZnO thin films, a gradual decrease in the photocurrent under UV illumination was observed because the amount of readsorbed oxygen molecules combined with free electrons was greater than that of the photogenerated electrons, and the defect sites of amorphous ZnO thin films also interrupted the movement of free electrons, causing a decrease in the photocurrent. But, ZnO thin films annealed by using the TDA method show a rapid increase and decrease in the photocurrent, and a stable photocurrent occurred during the repetitive on and off cycling of the UV light. This was due to a high surface-to-volume ratio that increased the amount of captured oxygen ions on the surface of the ZnO thin films and the improved crystallinity of the ZnO thin films through crystallization.

Furthermore, to investigate the photoresponse speed of the ZnO thin films with annealing process, we calculated the rise and decay time constant of the photocurrents by using bi-exponential fit. The following two equations were used for the rise and decay time constants of the photocurrent:^37^1
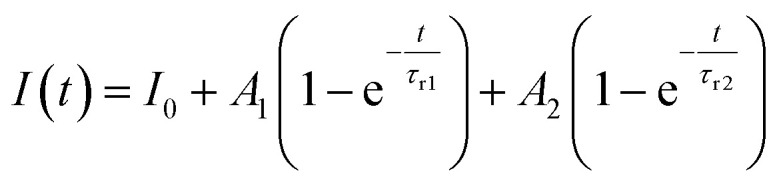
2
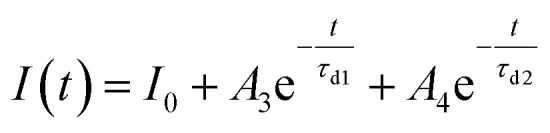
where *I*_0_ is the dark current; *A*_1_, *A*_2_, *A*_3_, and *A*_4_ are positive constants; *τ*_r1_ and *τ*_r2_ are the rise time constants; and *τ*_d1_ and *τ*_d2_ are the decay time constants. The estimated rise (*τ*_r1_ and *τ*_r2_) and decay (*τ*_d1_ and *τ*_d2_) time constants of the annealed ZnO thin film were 0.69 s and 2.58 s and 0.48 s and 2.44 s, respectively. As shown in Fig. 6(c), the increase in the photocurrent consisted of fast and slow steps, and 90% of the increase in the photocurrent was a rapid. The rapid increase in the photocurrent corresponded to the accumulation of photogenerated electrons under UV exposure, whereas the slow increase in the photocurrent was attributed to the readsorption of oxygen ions on the ZnO thin film surface. In other words, the ZnO thin films annealed with the TDA method had a high surface-to-volume ratio that allowed the ZnO thin films to quickly reach an equilibrium state of oxygen ion desorption and readsorption. The photocurrent decay also consisted of a fast and slow process, and 94.16% of the photocurrent decay was a fast process that was attributed to the recombination of photogenerated electron–hole pairs. The gradual decay of the photocurrent was attributed to the oxygen molecules gradually re-adsorbing on the surface of the ZnO thin films by reacting with free electrons. However, if defects (such as oxygen vacancies and interstitial zinc) existed in the ZnO thin films, the electrons would be trapped in defects, causing a slow rise and decay of the photocurrent. Table 1 summarizes reported study on ZnO-based transparent and flexible UV photodetectors and gives a comparison to the ZnO thin films annealed by using TDA method. As compared to most of the previous study, the transparent and flexible UV photodetectors which are prepared by using ZnO thin films annealed by using TDA method show the best performances in terms of fastest rise/decay times at lowest bias voltage. Ko *et al.* reported the highest value of dark current and photocurrent for their UV photodetectors, but they measured the photocurrent at relatively high bias voltage.^38^ But, in general, the value of dark current and photocurrent is affected to the value of bias voltage and it means that accurate comparison of the UV photodetector performance is impossible at different bias voltage. Thus, we compared the rise and decay time constant of UV photodetector, which is not affected to the bias voltage. In comparison to previous studies, our ZnO-based photodetectors are superior in terms of the fastest rise and decay time constant. We would also like to emphasize that the ZnO-based photodetectors based on TDA annealed ZnO thin films has high increment of photocurrent at lowest bias voltages. Therefore, the TDA method can improve the rise and decay speed of photocurrent by forming a large surface area and reducing the number of defects in the ZnO thin films. Additionally, the above results confirmed that the TDA method is an effective route for the fabrication of transparent and flexible photodetectors with fast response, high photosensitivity, high photoresponsivity, and great photocurrent stability.

**Table 1:** The performance of ZnO-based transparent and flexible UV photodetector

Sample	Dark current	Photocurrent	Rise time constant (*τ*_r1_)	Rise time constant (*τ*_r2_)	Decay time constant (*τ*_d1_)	Decay time constant (*τ*_d2_)	Bias voltage	Ref.
Graphene/ZnO	310 μA	518 μA	24.89 s	—	30.65 s	—	3 V	38
ZnO/GO	0.4 μA	1.5 μA	11.2 s	—	81 s	—	5 V	39
ZnO/graphene QDs	0.0221 μA	0.9205 μA	69.73 s	—	80.94 s	—	2 V	40
ZnO nanoneedle	20 nA	300 nA	27 s	27 s	7 s	40 s	0.3 V	41
ZnO thin films	0.871 μA	1.85 μA	0.69 s	2.58 s	0.48 s	2.44 s	0.1 V	This work

## 4. Conclusions

In conclusion, we deposited ZnO thin films onto PEN substrates by using the sol–gel spin-coating method and annealed the ZnO thin films with the TDA method which eliminated heat from the substrates. In contrast to existing annealing method for transparent and flexible ZnO thin films, the TDA method can anneal the ZnO thin films deposited onto polymer substrates without destroying the substrate. The morphology of the ZnO thin films annealed by using the TDA method changed from wrinkled network structure to a graphene-like nanosheet and the crystal phase also changed from amorphous to that of a crystalline. In addition, the ZnO thin films annealed by the TDA method showed improved UV photodetector performance, such as excellent stability and reproducibility, fast rise and decay time constants, high on/off ratio, and high photosensitivity and photoresponsivity. This indicates that the TDA method is an effective route for the fabrication of transparent and flexible UV photodetectors with a fast response speed, high photosensitivity and photoresponsivity, and great photocurrent stability. Therefore, the TDA method is a feasible alternative route for the fabrication of next generation wearable devices.

## Conflicts of interest

There are no conflicts to declare.

## Supplementary Material

RA-011-D0RA09869H-s001
