# Supplementary material for: Crystallization of ZnO thin films without polymer substrate deformation via thermal dissipation annealing method for next generation wearable devices
Source: RSC Adv. 2021 Jan 4;11(2):876–82. doi: 10.1039/d0ra09869h (PMC8693368; doi:10.1039/d0ra09869h)
Supplement: RA-011-D0RA09869H-s001 [file RA-011-D0RA09869H-s001.pdf]

Electronic Supplementary Material (ESI) for RSC Advances.
This journal is © The Royal Society of Chemistry 2020

Electronic Supplementary Information

Crystallization of ZnO thin films without polymer substrate deformation via thermal dissipation annealing method for next generation wearable devices

Dongwan Kim[1] and Jae-Young Leem[1,*]

[1]Department of Nanoscience & Engineering, Inje University, 197, Inje-ro, Gimhae-si, Gyeongsangnam-do 621-749, Republic of Korea

*Corresponding authors.

E-mail addresses: jyleem@inje.ac.kr

Tel.: +82-55-320-3716. Fax: +82-55-320-3631

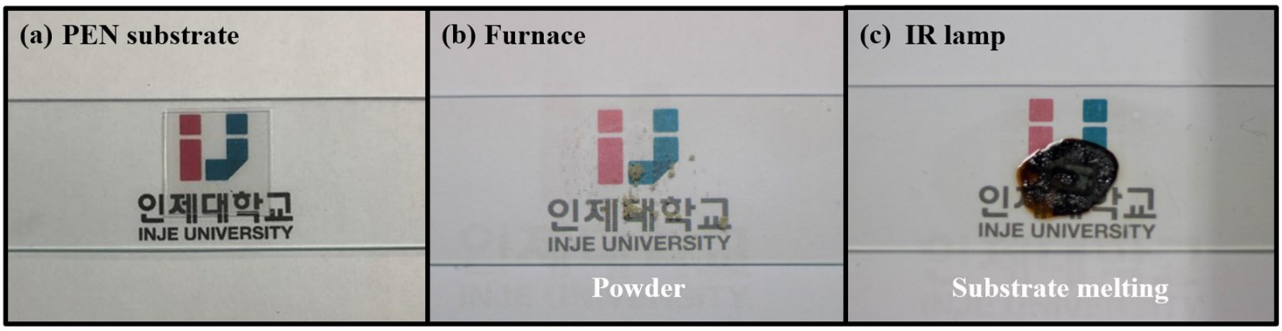

Fig. S1. Photographic image of (a) PEN substrate and sol-gel spin-coated ZnO thin films annealed by using (b) furnace and (c) IR lamp without dissipation system.
